# Prefrontal Nectin3 Reduction Mediates Adolescent Stress-Induced Deficits of Social Memory, Spatial Working Memory, and Dendritic Structure in Mice

**DOI:** 10.1007/s12264-020-00499-2

**Published:** 2020-05-08

**Authors:** Hong-Li Wang, Ji-Tao Li, Han Wang, Ya-Xin Sun, Rui Liu, Xiao-Dong Wang, Yun-Ai Su, Tian-Mei Si

**Affiliations:** 1 National Clinical Research Center for Mental Disorders (Peking University Sixth Hospital/Institute of Mental Health) and the Key Laboratory of Mental Health, Ministry of Health (Peking University Sixth Hospital), Beijing, 100191 China; 2 The National Clinical Research Center for Mental Disorders & Beijing Key Laboratory of Mental Disorders, Beijing Anding Hospital, Capital Medical University & the Advanced Innovation Center for Human Brain Protection, Capital Medical University, Beijing, 100088 China; 3 Department of Neurobiology, Key Laboratory of Medical Neurobiology of The Ministry of Health of China, Zhejiang Province Key Laboratory of Neurobiology, Zhejiang University School of Medicine, Hangzhou, 310003 China

**Keywords:** Adolescence, Chronic stress, Cell adhesion molecule, Prefrontal cortex, Social memory

## Abstract

Chronic stress may disrupt the normal neurodevelopmental trajectory of the adolescent brain (especially the prefrontal cortex) and contribute to the pathophysiology of stress-related mental illnesses, but the underlying molecular mechanisms remain unclear. Here, we investigated how synaptic cell adhesion molecules (e.g., nectin3) are involved in the effects of adolescent chronic stress on mouse medial prefrontal cortex (mPFC). Male C57BL/6N mice were subjected to chronic social instability stress from postnatal days 29 to 77. One week later, the mice exposed to chronic stress exhibited impaired social recognition and spatial working memory, simplified dendritic structure, and reduced spine density in the mPFC. Membrane localization of nectin3 was also altered, and was significantly correlated with behavioral performance. Furthermore, knocking down mPFC nectin3 expression by adeno-associated virus in adolescent mice reproduced the stress-induced changes in behavior and mPFC morphology. These results support the hypothesis that nectin3 is a potential mediator of the effects of adolescent chronic stress on prefrontal structural and functional abnormalities.

**Electronic supplementary material:**

The online version of this article (10.1007/s12264-020-00499-2) contains supplementary material, which is available to authorized users.

## Introduction

The prefrontal cortex (PFC) has long been implicated in the pathophysiology of stress-related psychiatric disorders such as depression and schizophrenia [1, 2]. As a late-maturing brain region, the PFC undergoes extensive structural remodeling during adolescence and early adulthood [3], including the pruning of initially over-produced excitatory synapses [4], and the maturation of inhibitory synapses [5]. Accompanying the structural development is the functional maturation of PFC-dependent behaviors, such as social behaviors and working memory [6, 7]. Chronic stress during adolescence has been shown to alter prefrontal dendritic architecture and to impair cognitive and social functions [8–10], but the neurobiological mechanisms have just started to be uncovered.

Synaptic cell adhesion molecules (CAMs) contain several families of proteins, such as N-cadherin, catenins, nectins, and neuroligins [11, 12]. These molecules are located at adherens and/or synaptic junctions, forming inter-neuronal connections and dynamically shaping synaptic plasticity by modulating synapse formation, maturation, and transmission [13–15]. Several CAMs are altered by stress, such as nectin3 [16, 17], nectin1 [18, 19], neuroligin2 [10, 20], β-catenin [21–23], and N-cadherin [22, 24]. Evidence also supports mediating roles of CAMs in the effects of stress on behavior and dendritic structure [16, 20, 25, 26], but the majority of these studies have focused on the hippocampus and postnatal/adult stress [16, 20, 25]. Only one study has reported the involvement of prefrontal neuroligin2 expression in adolescent stress-induced attention deficits [10]. Therefore, it remains unknown whether and how prefrontal CAM expression is associated with the behavioral and structural abnormalities induced by adolescent chronic stress.

In this study, we investigated the involvement of CAMs in chronic-stress-induced effects on the medial PFC (mPFC) in adolescent mice. Specifically, using the adolescent chronic social instability stress paradigm [27, 28], we first evaluated how adolescent chronic stress would affect mPFC-dependent social and spatial working memory behaviors and the mPFC dendritic architecture. We then tested the CAM involvement in these stress effects by examining whether adolescent stress would alter mPFC CAM expression and whether manipulating prefrontal CAM expression *via* adeno-associated virus (AAV) could reproduce the behavioral and structural consequences of chronic stress exposure.

## Materials and Methods

### Animals

Adolescent male C57BL/6N mice (*n* = 72, 21 ± 1 days old; Vital River Laboratories, Beijing, China) were housed in groups of 4 per cage under a 12L:12D cycle (lights on at 08: 00) and at a constant temperature (23 ± 1 °C) with free access to food and water. All experiments were carried out in accordance with the National Institute of Health’s Guide for the Use and Care of Laboratory Animals and were approved by the Peking University Committee on Animal Care and Use.

### Chronic Social Instability Stress

**Fig. 1:**
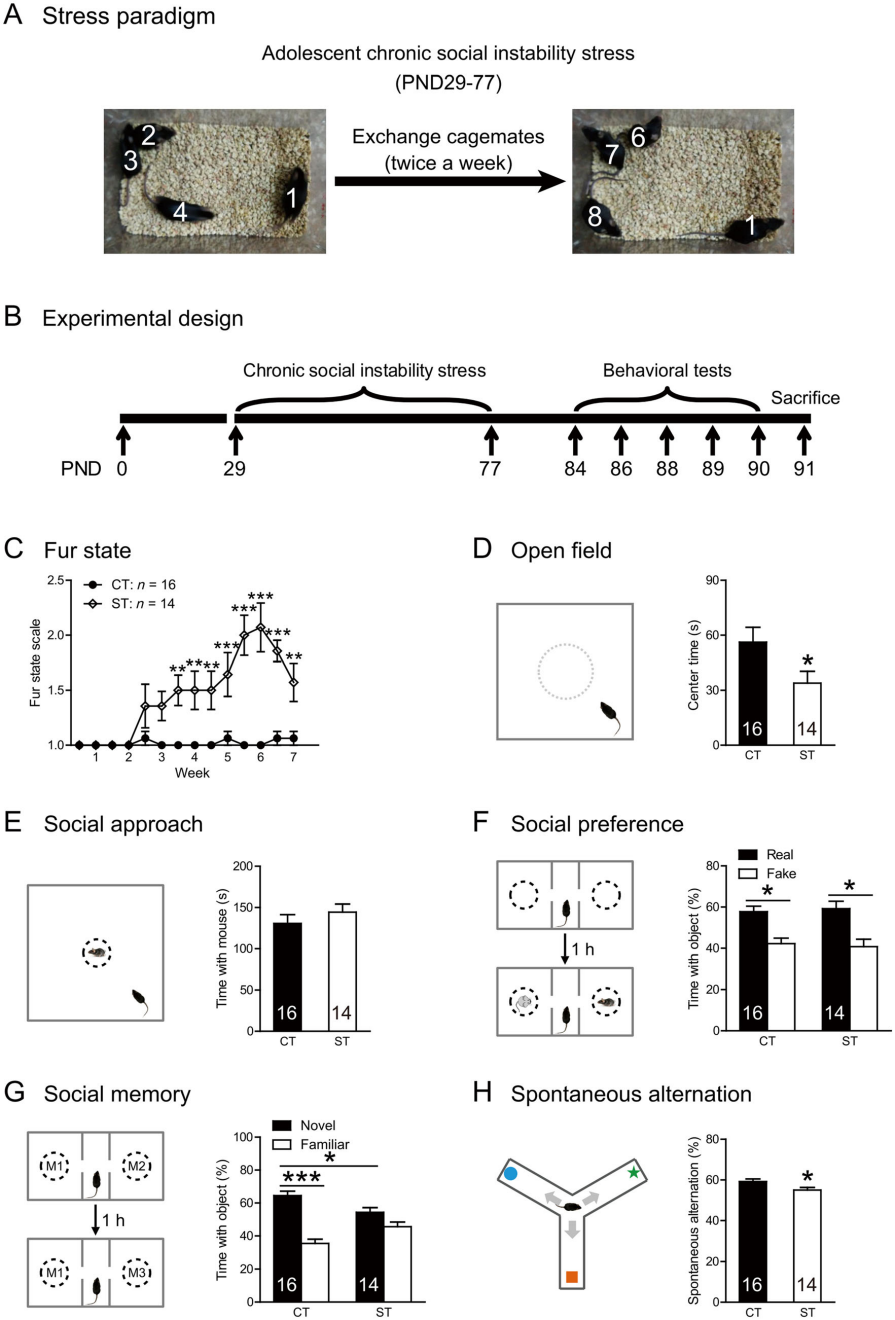
The paradigm, experimental design, and behavioral effects of adolescent chronic social instability stress. **A** The paradigm of adolescent chronic social instability stress. **B** The experimental timeline of the behavioral procedure and brain tissue acquisition after stress exposure. **C** The fur states of stressed mice worsen throughout stress exposure. **D** Adolescent stress increases the anxiety levels in the open field. **E**–**G** Adolescent stress does not affect social approach (**E**) and preference (**F**), but impairs social memory (**G**). **H** Stressed mice have a lower spontaneous alternation ratio in the Y-maze test than control mice. CT, control; ST, stress. Numbers in each bar indicate the number of animals in each group. Data represent the mean ± SEM. *P < 0.05; **P < 0.01; ***P < 0.001. PND, postnatal day.

The chronic social instability stress paradigm was carried out as described previously [27] (Fig. 1A). After habituation in the vivarium for 7 days after arrival, mice were randomly divided into stress (ST) and control (CT) groups. In the ST group, cage-mates were changed twice a week for 7 weeks (postnatal days 29–77). To prevent the mice from developing stable social hierarchies, at each rotation 4 animals were regrouped in a cage such that an individual mouse was randomly introduced to 3 experimental mice that it had not encountered for at least one week. Cage-mates in the CT group remained unchanged. At the end of the chronic stress procedure, mice in both groups were separated and singly housed for 7 days and then subjected to behavioral tests. Body weight and fur state were monitored 24 h after each change of cage-mates.

Fur state was scored independently by two experimenters on a 4-point scale (1, normal condition; 4, worst condition) [27, 29]. Three body parts were examined (body fur, whiskers, and conjunctivae). The scoring scheme was as follows: (1) smooth and shiny fur with no tousled and spiky patches; whiskers and conjunctivae normal; (2) slightly fluffy fur with some spiky patches; whiskers and conjunctivae normal; (3) fluffy fur on most of the body, slightly stained; whiskers and conjunctivae slightly abnormal; and (4) fluffy fur with some bald or wound patches; whisker lost and conjunctivae congested.

### Experimental Design

This study contained two experiments. In Experiment 1 (Fig. 1B), we investigated the behavioral, morphological, and molecular effects of adolescent chronic stress. A total of 32 mice (16/group) were used for a series of behavioral tests (open field, social approach, social preference, social memory, and spontaneous alternation in the Y maze). Note that two mice in the stressed group died during stress exposure, leaving 14 mice for further analyses. On day 91 (24 h after the last behavioral test), each mouse was decapitated after anesthesia and the brain removed for morphological analyses (*n* = 5/group) and western blot (*n* = 6/group).

In Experiment 2, we investigated whether knocking down the prefrontal nectin3 expression (nectin3-KD) could mimic the effects of stress on behavior and mPFC morphology (Fig. 5A). A total of 35 mice were used (16 controls and 19 nectin3-KD mice). To match the stress schedule, AAV was injected into the mouse mPFC on day 29. Behavioral tests and neurobiological analyses were performed at the same time points as in Experiment 1. The sample sizes for the two experiments were based on previous studies [16, 25].

### Behavioral Tests

All of the behavioral tests were carried out between 09:00 and 16:00 as described previously [28, 30, 31]. Behavior in the open field was analyzed using ANY-maze 4.98 (Stoelting, Wood Dale, IL). The social tests and Y-maze spontaneous alternation task were scored by an experimenter who was unaware of the aims of the study.

#### Open Field

The open field test was performed in an arena (50 cm × 50 cm × 50 cm, illuminated at 60 lux) made of gray polyvinyl chloride. During the test, a mouse was put in one corner and freely explored the arena for 10 min. To avoid residual olfactory traces, we cleaned the arena with 75% ethanol after each test. Using the ANY-maze software, we analyzed the following variables: total distance traveled during the test, time spent in the center zone (20 cm in diameter), and latency to the center zone. The number of fecal pellets was recorded by the experimenter as another index to reflex animals’ anxiety levels.

#### Social Approach

This test was conducted in an open compartment made of gray polyvinyl chloride (50 cm × 50 cm × 50 cm, illuminated at 15 lux). A wire-mesh cylinder was fixed in the center with a stimulus mouse inside (male, 12 weeks old). During the test, another mouse was put in one corner of the compartment and allowed to interact with the stimulus mouse for 10 min. The time spent interacting with the stimulus mouse was recorded manually. Video tracking software was used to measure the latency to the central zone (15 cm in diameter).

#### Social Preference

Social preference was tested in a three-chambered box (50 cm × 25 cm × 50 cm) containing two side-compartments (20 cm × 25 cm × 50 cm), each with a wire-mesh cylinder in the center. During the acquisition session, each mouse was allowed to freely explore the box with empty cylinders for 10 min. An hour later, a fake mouse was put into one cylinder and a real mouse was put into the other. During the test trial, the test mouse was placed in the center chamber and allowed to habituate for 1 min. Then the doors to the side compartments were opened and the mouse allowed to freely explore the chambers for 10 min. A trained experimenter blind to the experimental conditions manually recorded the time the mouse spent sniffing each target (real *versus* fake mouse). The preference index was calculated as the time spent with each target divided by the total exploration time × 100%.

#### Social Memory

In the three-chambered box, the test started with a 10-min acquisition session, during which there was a male mouse in each cylinder and the test mouse was allowed to freely interact with them. The test session was carried out 1 h later, when the mouse in one cylinder was replaced by a new counterpart and the test mouse was given 10 min to interact with the two mice (one familiar and the other novel). The times spent interacting with the novel and familiar mice were recorded. The discrimination index was defined as the time spent with each mouse divided by the total exploration time × 100%.

#### Spontaneous Alternation

The Y-maze apparatus had three arms (30 cm × 10 cm × 15 cm, made of gray polyvinyl chloride), with the same angle (120°) between each of the arms. Intra-maze cues (blue circle, orange square, and green star) and some external cues were provided. The maze was illuminated at 15 lux during the test. Each mouse was placed in the center of the maze and allowed to freely explore the three arms for 8 min. The total distance traveled, the number of arm entries, and the spontaneous alternation ratio were recorded.

### Golgi-Cox Staining and Morphological Analysis

Mice were transcardially perfused with 0.9% saline under anesthesia (pentobarbital sodium, 200 mg/kg, i.p.). The brains were quickly removed and immersed in Golgi-Cox solution for 2 weeks in the dark at room temperature. Then the brains were rinsed in 20 mL ddH_2_O, transferred to 20 mL 30% sucrose, and left in the dark for 5 days. Serial coronal sections were cut at 120 μm on a Microm HM vibratome (Thermo Scientific, Walldorf, Germany) and collected in 6% sucrose in ddH_2_O at 4 °C. The sections were then mounted on Superfrost plus slides (Thermo Scientific) and pre-processed according to a previously-described protocol [17, 32].

Pyramidal neurons in superficial (II/III) and deep (V) layers of the mPFC, fully impregnated with Golgi stain and minimally overlapped with other stained cells, were used for morphological analyses (6–8 neurons from each layer in each subregion per mouse; the number of neurons was chosen based on previous studies [16, 32]). For each neuron, the circumference of the cell body and the dendritic branches in the x, y, and z directions were manually traced at ×400 with Neurolucida software (MicroBrightField Bioscience, Williston, VT).

Sholl analysis was used to assess the total dendritic length and dendritic complexity using NeuroExplorer software. For each neuron, the dendritic length was measured as the sum of the lengths of the apical main dendrite and branches. The apical main dendrite refers to the large apical dendrite that connects the soma to distal tuft dendrites. The apical branches included all the oblique dendrites that emanated from the apical main dendrite. Dendritic complexity was measured as the number of intersections per concentric circle (20 μm) of increasing distance from the cell body.

For dendritic spine analysis of mPFC pyramidal neurons, the inclusion criteria for segments to be analyzed were as follows [32]: (1) segments of apical main or oblique dendrites initiated at 120 µm from the soma; (2) segment length >  30 µm; (3) comparable segment diameter for each dendritic domain; and (4) no overlap with other segments, to prevent possible confusion in spine visualization. Bright-field z-series images of dendritic segments (6–8 segments from each dendritic domain per animal) were digitized at ×1000 using a CoolSNAP MP5 CCD camera (Roper Scientific, Tucson, AZ) mounted to an Olympus BX51 microscope (Tokyo, Japan). Dendritic spines in the mPFC were classified as thin, stubby, or mushroom types according to the criteria in the literature [33], and counted using NIH ImageJ software. Spine density is expressed as the number of spines per 10 µm of dendrite.

### Western Blot

Based on a previous description [17], mice were anesthetized with isoflurane-O2 (4–5:100). The mPFC was rapidly dissected from the brain on ice, homogenized in ice-cold lysis buffer (137 mmol/L NaCl, 20 mmol/L Tris–HCl (pH 8.0), 1% NP-40, 10% glycerol, 1 mmol/L phenylmethylsulfonyl chloride, 10 mg/mL aprotinin, 1 mg/mL leupeptin, and 0.5 mmol/L sodium vanadate), sonicated, and centrifuged. The supernatants were stored at − 80 °C until use. Membrane proteins were collected using the ProteoExtract^®^ Subcellular Proteome Extraction Kit (Cat#: 539790, Calbiochem, Darmstadt, Germany) according to the manufacturer’s instructions. The mPFC of one hemisphere was used to assess total proteins, and that of the other hemisphere was used to measure membrane expression levels.

We prepared 5% sodium dodecyl sulfate polyacrylamide gel electrophoresis laminated gels and 10% separating gels for electrophoresis. Samples containing 20 µg protein were added to the gels and transferred electrophoretically to polyvinylidene difluoride membranes (Millipore, Bedford, MA). The membranes were then blocked with 5% non-fat milk diluted in Tris-buffered saline-Tween (TBST; containing 150 mmol/L NaCl, 10 mmol/L Tris-HCl, and 0.1% Tween, adjusted to pH 7.5) for 1 h at room temperature and labeled overnight at 4 °C with primary antibodies diluted in TBST containing 5% non-fat milk (nectin1: 1:5000, sc-28639, Santa Cruz; nectin3: 1:5000, sc-28637, Santa Cruz; N-cadherin: 1:5000, ab18203, Abcam; β-catenin: 1:50000, 281003, Synaptic Systems; neuroligin1: 1:10000, 129013, Synaptic Systems; neuroligin2: 1:10000, 129203, Synaptic Systems; GAPDH: 1:20000, 2118, Cell Signaling; Na, K-ATPase: 1:10000, 3010, Cell Signaling; β-actin: 1:20000, 3700S, Cell Signaling). After 2-h incubation with horseradish peroxidase-conjugated secondary antibodies (1:5000–10000, Zhongshan Gold Bridge Biotechnology, China, diluted in TBST) at room temperature, bands were visualized using the Amersham Imager 600 (GE Healthcare, PA) and analyzed using Quantity One 4.2 (Bio-Rad, Hercules, CA) by an investigator blind to the treatment conditions. The values were corrected based on their corresponding control protein levels. All results were normalized by taking the value of the vehicle group as 100%.

### Immunofluorescence and Image Analysis

Mice were anesthetized with sodium pentobarbital (200 mg/kg, i.p.) and then transcardially perfused with 0.9% saline followed by 4% paraformaldehyde in 0.1 mol/L PBS. The brains were removed, post-fixed for 12 h in the same fixative, and then stored in 30% sucrose for 3 days at 4 °C. Next, the brains were quenched in N-hexane at − 60 °C and stored at − 80 °C until use. Following cryoprotection, serial coronal sections through the mPFC (1.98 mm–1.54 mm from bregma) were cut at 30 μm on a cryostat (Leica, Wetzlar, Germany). Sections at 180-μm intervals were blocked with 1% normal donkey serum for 1 h at room temperature and then incubated overnight at 4 °C with primary antibodies (nectin3: 1:1000, ab63931; nectin1: 1:1000; ab66985; NeuN: 1:5000, ab104225, all from Abcam, Cambridge, UK). The next day, the sections were rinsed 3 times in 0.1 mol/L PBS and then labeled with Alexa Fluor 594-conjugated secondary antibodies (1:500; Invitrogen, Carlsbad, CA) for 3 h at room temperature. The sections were rinsed, transferred onto slides, and coverslipped with Vectashield containing 4′,6-diamidino-2-phenylindole (DAPI; Vector Laboratories, Burlingame, CA).

The sections were assigned random numbers for analyses. Images were acquired from 4 sections per animal at 100× or 200× using an Olympus IX71 microscope equipped with a charge-coupled device camera (CoolSNAP MP5, Roper Scientific Corp.). ImageJ software (NIH) was used to quantify the immunoreactivity of nectin1 and nectin3 and the density of NeuN-positive cells. Relative protein levels were calculated as differences in optical density between the mPFC and the corpus callosum (background). Results were normalized by taking the mean value of the control group as 100%. For co-localization analysis, images (1024 × 1024 pixel^2^) were obtained at 200× (optical section thickness, 0.362 μm) or 600× (optical section thickness, 0.196 μm) using an Olympus IX81 laser-scanning confocal microscope (Olympus, Tokyo, Japan) with the Kalman filter and sequential scanning mode under identical settings for laser power, photomultiplier gain, and offset. The brightness and contrast of images were optimized using FV10-ASW 1.7 software (Olympus).

### Stereotactic Surgery and Viral Microinjection

The adeno-associated virus (AAV) 2/8 vectors that were generated and purified by Obio Technology (Shanghai, China) were used to suppress the nectin3 protein levels. The short hairpin RNA (shRNA) sequence for nectin3 was 5′-TGTGTCCTGGAGGCGGCAAAGCACAACTT-3′. We used two types of virus: AAV-shNectin3 (AAV2/8-CMV-Nectin3.shRNA-terminator-CAG-EGFP-WPRE-BGH-polyA, 3.9 × 10^12^ viral genomes/mL) and control virus (AAV2/8-CMV-scrambled.shRNA-terminator-CAG-EGFP-WPRE-BGH-polyA, 3.5 × 10^12^ viral genomes/mL).

The procedures for stereotaxic surgery and microinjection were as previously described. After 7 days of habituation, adolescent mice (29 days old) were anesthetized (1.5%–1.8% isoflurane in 1 L/min air) with perioperative meloxicam analgesia (3 mg/kg, i.p.) and received viral microinjections into the bilateral mPFC (0.5 µL per side, 0.1 µL/min) through a glass micropipette. The injection coordinates (relative to bregma) were anterior + 1.8 mm, lateral ± 0.4 mm, and ventral − 1.8 mm. The micropipette was left in the site for a further 5 min. Mice were allowed to recover until the beginning of behavioral tests (day 84).

### Fluorescent mRNA *In Situ* Hybridization

The mPFC mRNA expression was visualized using RNAscope (Advanced Cell Diagnostics). Following the manufacturer’s guidelines, mouse brains were quickly dissected, dehydrated, and frozen. After cryoprotection, serial coronal sections through the mPFC (1.98 mm–1.54 mm from bregma) were cut at 15 μm on a cryostat (Leica). Slides at 180 μm intervals were dried at − 20 °C for 1 h and then stored at − 80 °C for up to one week. The slides were processed following the RNAscope protocol using a fluorescent multiplex reagent kit (ACD, catalog #323100) and probes for *Pvrl3* (Mm-Pvrl3-C1; ACD, catalog #300031), *Gad1* (Mm-Gad1-C2; ACD, catalog #400951), and *Slc17a7* (Mm-Slc17a7-C3; ACD, catalog #416631).

For co-localization analysis, images (1024 × 1024 pixel^2^) were captured at 200× (optical section thickness, 5.4 μm) or 400× (optical section thickness, 2.64 μm) using a Nikon A1RHD25 laser-scanning confocal microscope (Tokyo, Japan). Images were then separated into multiple color channels and cell nuclei were identified in the DAPI channel. Signals in the red, green, and magenta channels were thresholded, identified, and filtered by the locations of nuclei. If a signal was found in a nucleus, the cell was defined as “positive” for the respective RNA species. Nuclei positive for *Gad1* or *Slc17a7* were finally filtered to determine whether they co-expressed nectin3.

### Statistical Analyses

Data are presented as the mean ± standard error of the mean (SEM) in the figures. Independent samples *t*-tests were used for group comparisons. For body weight, fur state, and dendritic intersection, repeated measures analysis of variance was used, with time or distance as a within-subject factor and treatment as a between-subject factor, followed by the Bonferroni *post hoc* test when appropriate. The association between behavioral performance and fur state or CAM expression was quantified using Pearson’s correlation. Differences with *P* < 0.05 were considered statistically significant.

## Results

### Adolescent Chronic Social Stress Impairs Social Memory and Spatial Working Memory

Adolescent chronic social stress had minimal influence on body weight (main effect of treatment: *F*_(1, 27)_ = 0.177, *P* = 0.678, Fig. S1A), but caused the fur state to deteriorate in the stressed group (Fig. 1C; main effect of treatment: *F*_(1, 28)_ = 47.48, * P *< 0.0001; treatment × time interaction: *F*_(13, 364)_ = 7.576, *P* < 0.0001), especially after the third week of stress exposure (all *t* > 3.553, Bonferroni-corrected all *P* < 0.01). Adolescent stress exposure also increased the anxiety-like behaviors in the open field (*t*_(28)_ = 2.096, *P* = 0.045, Figs. 1D and S1B). These results are consistent with previous findings using this paradigm [27].

Adolescent chronic stress did not significantly alter social approach and social preference. The stressed and control animals spent comparable times interacting with the stimulus mouse behind wire mesh in the social approach test (*t*_(28)_ = 0.871, *P* = 0.391; Figs. 1E and S1C). Both groups exhibited clear social preference, as they spent significantly more time interacting with the real mouse than the fake one (CT: *t*_(15)_ = 2.899, *P* = 0.011; ST: *t*_(13)_ = 2.580, *P* = 0.023; Figs. 1F and S1D), and their preference indices were comparable (*t*_(28)_ = 0.338, *P* = 0.738).

In contrast, stressed animals exhibited impairments in the social memory test and spatial working memory assessed in the Y maze. In the social memory test (Fig. 1G), while control mice distinguished a novel from a familiar mouse (*t*_(15)_ = 5.520, *P* < 0.001), stressed mice failed to do so (*t*_(13)_ = 1.558, *P* = 0.143) and spent a significantly lower percentage of time interacting with a novel mouse compared with controls (*t*_(28)_ = 2.633, *P* = 0.014). The exploration time during the acquisition session was comparable in the two groups (Fig. S1E). In the Y-maze task, stressed animals had lower spontaneous alternation ratios than controls (*t*_(28)_ = 2.389, *P* = 0.024; Fig. 1H), despite the similar numbers of entries in the two groups (Fig. S1F).

Deterioration of fur state has been used as an indicator of stress experiences [34]. To determine whether behavioral alterations in the stressed group directly result from stress exposure, we correlated their fur state ratings with behavioral performances and found significant correlations in various behavioral measures (all *r* > 0.532, all *P* < 0.05, Fig. S2), confirming the association between stress adversity and behavioral changes.

### Adolescent Chronic Social Stress Simplifies Apical Dendritic Structure and Decreases Spine Density of Pyramidal Neurons in Mouse mPFC

**Fig. 2:**
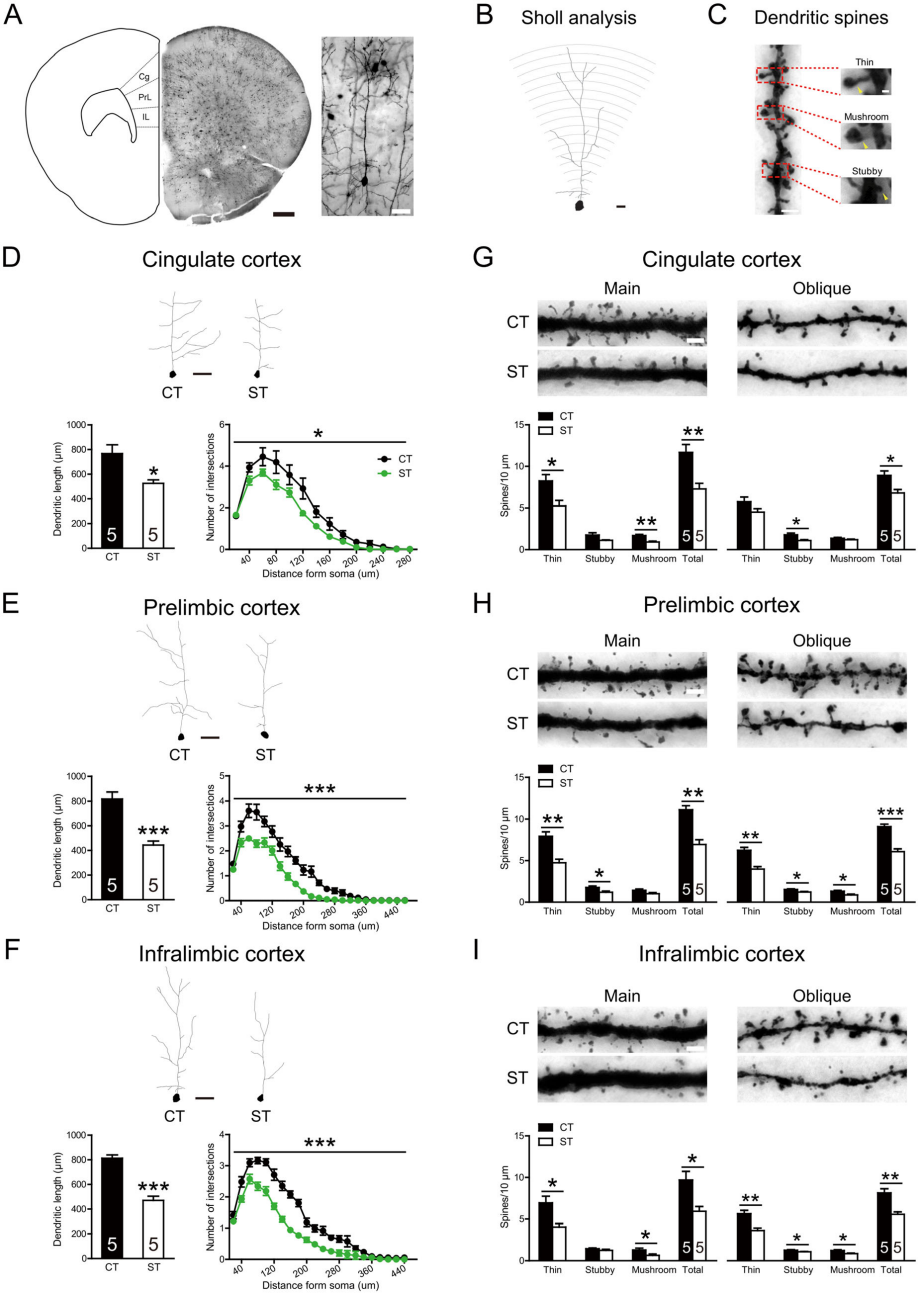
Effects of adolescent chronic social instability stress on the dendritic architecture of the mouse medial prefrontal cortex (mPFC). **A** Diagrams of a coronal section (left) showing three regions of interest [dashed area; cingulate (Cg), prelimbic (PrL) and infralimbic (IL) cortex], a Golgi-stained coronal section (middle; scale bar, 500 μm), and a Golgi-impregnated mPFC pyramidal neuron from an adult mouse (right; scale bar, 50 μm). **B** Sholl analysis. Concentric circles (20 μm apart) are drawn on an apical dendrite. **C** Dendritic spines are categorized to three subtypes: thin (long, thin protrusions with a bulbous head), mushroom (protrusions with a small neck and a large head), and stubby (protrusions closely connected to the dendritic shaft and lacking a clear neck). **D**–**F** Adolescent stress reduces the length and complexity of apical dendrites of pyramidal neurons in cingulate (**D**), prelimbic (**E**), and infralimbic (**F**) cortex (scale bar, 50 μm). Six to eight neurons were analyzed for each layer in a given region per animal. **G**–**I** Adolescent stress reduces the spine density on apical main and oblique dendrites in cingulate (**G**), prelimbic (**H**), and infralimbic (**I**) cortex (scale bar, 5 μm). We analyzed 6–8 segments for each dendritic domain per animal. The total spine density was calculated as the sum of three spine types. CT, control; ST, stress. Numbers in each bar indicate the number of animals in each group. Data represent the mean ± SEM. *P < 0.05; **P < 0.01; ***P < 0.001.

We examined the dendritic structure of pyramidal neurons in three mPFC subregions [cingulate (Cg), prelimbic (PrL), and infralimbic (IL) areas] using the Golgi-Cox staining method (Fig. 2A–C). Compared with controls, stressed mice showed reduced length and complexity of apical dendrites in each of these subregions (Cg: length: *t*_(8)_ = 3.119, *P* = 0.014; complexity: *F*_(1, 8)_ = 5.772, *P* = 0.043, Fig. 2D; PrL: length: *t*_(8)_ = 5.845, *P* < 0.001; complexity: *F*_(1, 8)_ = 25.723, *P* = 0.001, Fig. 2E; IL: length: *t*_(8)_ = 7.828, *P* < 0.001; complexity: *F*_(1, 8)_ = 56.747, *P* < 0.001, Fig. 2F). We further analyzed the superficial (II/III) and deep (V) layers of pyramidal neurons in each subregion and found a similar reduction in both layer types (Fig. S3).

The number of spines was also reduced by adolescent chronic stress. Spine loss was significant in stressed mice on both the main (all *t*_(8)_ > 3.189, all *P* < 0.013, Fig. 2G–I, left panel) and oblique apical dendrites (all *t*_(8)_ > 3.055, all *P* < 0.016, Fig. 2G–I, right panel) in all three subregions. The spine loss occurred in more than one spine type in each subregion (all *t*_(8)_ > 2.337, all *P* < 0.048). Examination of superficial and deep layers showed a general reduction in stressed animals, with various spine types affected (Fig. S4).

Together, these results demonstrated that adolescent chronic stress simplified apical dendritic structure and reduced spine density throughout the mPFC. We also counted the number of neurons positive for neuronal nuclei antigen (NeuN^+^) in the three mPFC subregions (Fig. S5) and found comparable numbers in the stressed and control animals (*P* > 0.449), which indicated that the dendritic changes cannot be accounted for by neuronal loss.

### Adolescent Chronic Social Stress Specifically Alters Nectin3 Membrane Expression in Mouse mPFC

**Fig. 3:**
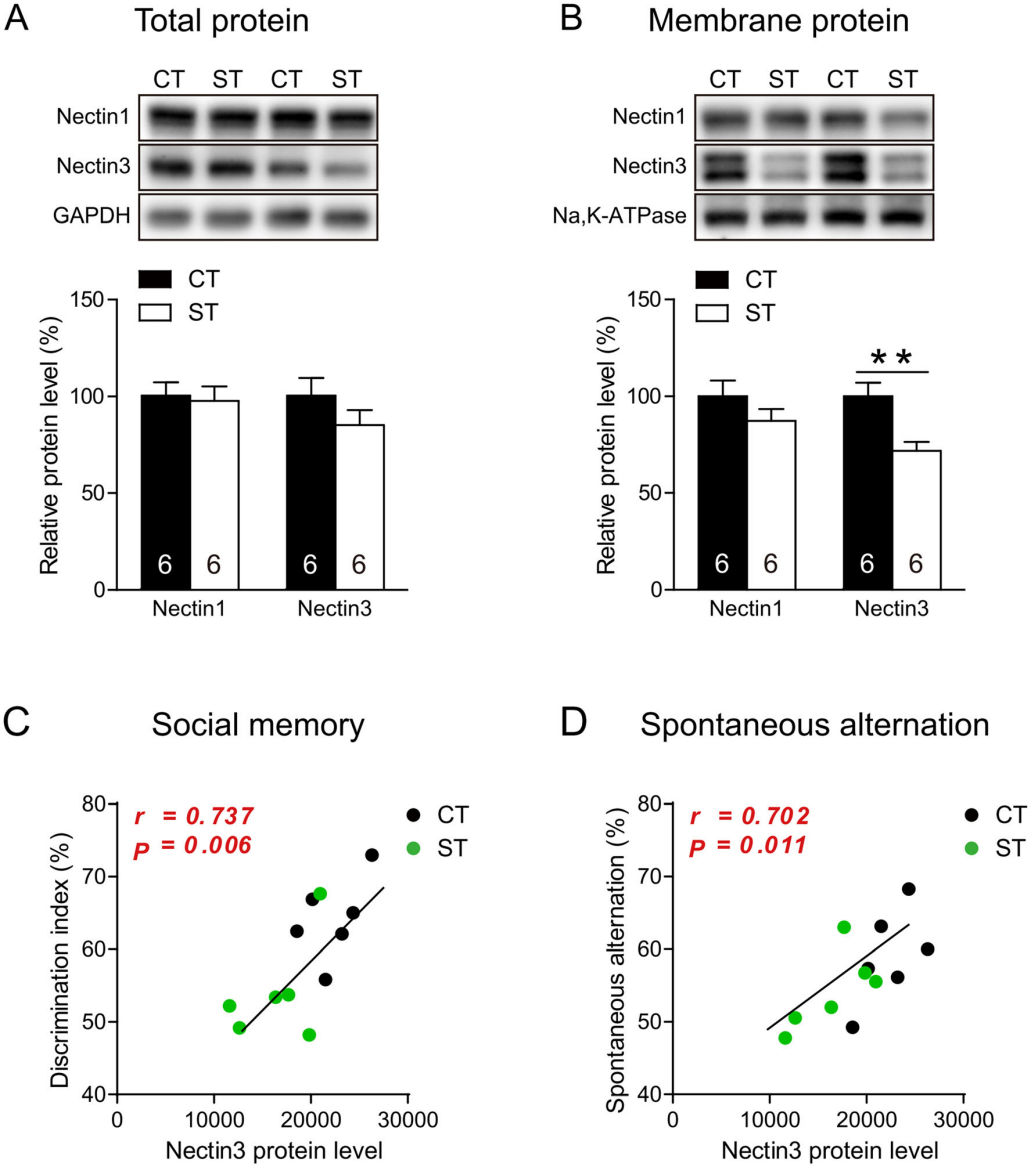
Effects of adolescent chronic social instability stress on nectin1 and nectin3 expression in mouse medial prefrontal cortex (mPFC). **A** Adolescent stress does not change the total protein expression of nectin1 and nectin3. **B** Adolescent stress significantly reduces the membrane protein levels of nectin3, but not nectin1. **C**, **D** Nectin3 membrane protein levels are significantly correlated with the discrimination index in the social memory test (**C**) and with the spontaneous alternation ratio in the Y maze (**D**). CT, control; ST, stress. Numbers in each bar indicate the number of animals in each group. Data represent the mean ± SEM. *P < 0.05.

In this experiment, we assessed whether and how adolescent chronic stress may influence the prefrontal expression of the synaptic CAMs nectin1, nectin3, N-cadherin, β-catenin, neuroligin1, and neuroligin2. At the total protein level (Fig. 3A), adolescent stress seemed to downregulate nectin1 and nectin3 expression, but the change did not reach statistical significance. At the membrane protein level (Fig. 3B), nectin3 was significantly reduced by adolescent chronic stress (*t*_(10)_ = 3.327, *P* = 0.008). The protein levels of the other CAMs were not affected at either the total or the membrane level (Fig. S6).

We then assessed the correlations between nectin3 membrane levels in the mPFC and behavioral performances. Nectin3 membrane expression did not correlate with anxiety-like behaviors, or performance in the social approach and preference tests (Fig. S7), but significantly correlated with the discrimination index in the social memory test (Fig. 3C) and with the Y-maze spontaneous alternation ratio (Fig. 3D), which indicates a possible association between nectin3 expression and stress-induced social and cognitive deficits.

### Nectin3 Knockdown During Adolescence Reproduces Adolescent Stress-Induced Social and Working Memory Deficits, Dendritic Atrophy, and Spine Loss

**Fig. 4:**
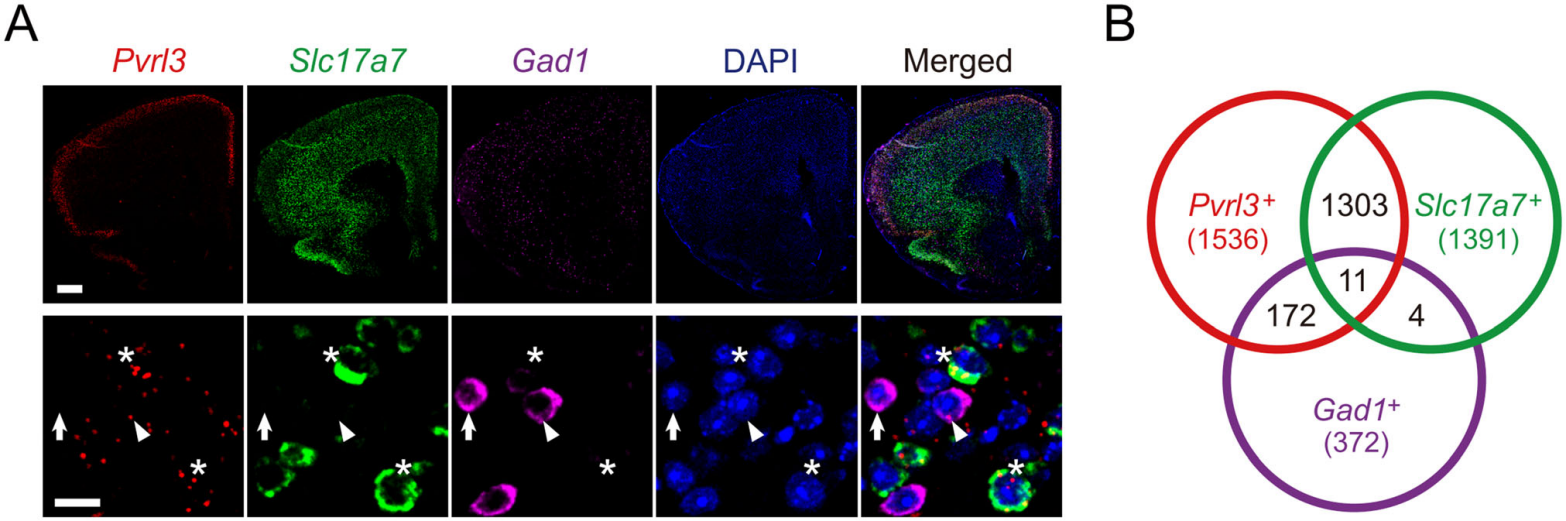
Nectin3 expression in excitatory and inhibitory neurons in mouse medial prefrontal cortex. **A** Representative (upper, scale bar, 500 μm), and magnified (lower, scale bar, 20 μm) images showing the mRNA expression of *Pvrl3*, *Slc17a7,* and *Gad1* (stars, neurons that co-express *Pvrl3* and *Slc17a7*; arrowheads, neurons that co-express *Pvrl3* and *Gad1*; arrows, *Gad1*-expressing cells without detectable *Pvrl3* expression). **B** Numbers of neurons that co-express *Pvrl3* and *Slc17a7*, *Pvrl3* and *Gad1, Slc17a7* and *Gad1*, and *Pvrl3*, *Slc17a7*, and *Gad1*. Numbers in parentheses indicate the total number of neurons expressing the corresponding mRNA.

To investigate how nectin3 downregulation may contribute to stress-induced behavioral and structural abnormalities, we examined the effects of adolescent nectin3 knockdown *via* AAV injection. We first confirmed that nectin3 mRNA (*Pvrl3*) is expressed in the mouse mPFC using the RNAscope technique (Figs. 4A and S8). The co-localization of *Pvrl3* with *Slc17a7* (the mRNA of vascular glutamate transporter 1, an excitatory neuron marker) and *Gad1* (the mRNA of GAD67, an inhibitory neuron marker) in the mPFC (Fig. 4B) revealed that the majority of *Pvrl3*^*+*^ neurons co-localized with *Slc17a7*^*+*^ neurons (1303 out of 1536 *Pvrl3*^*+*^ neurons, 84.83%, and out of 1391 *Slc17a7*^*+*^ neurons, 93.67%). Some *Pvrl3*^*+*^ neurons also co-localized with *Gad1*^*+*^ neurons (172 out of 1536 *Pvrl3*^*+*^ neurons, 11.20%, and out of 372 *Gad1*^*+*^ neurons, 46.24%), indicating that nectin3 is expressed in both excitatory and inhibitory neurons in mouse mPFC.

**Fig. 5:**
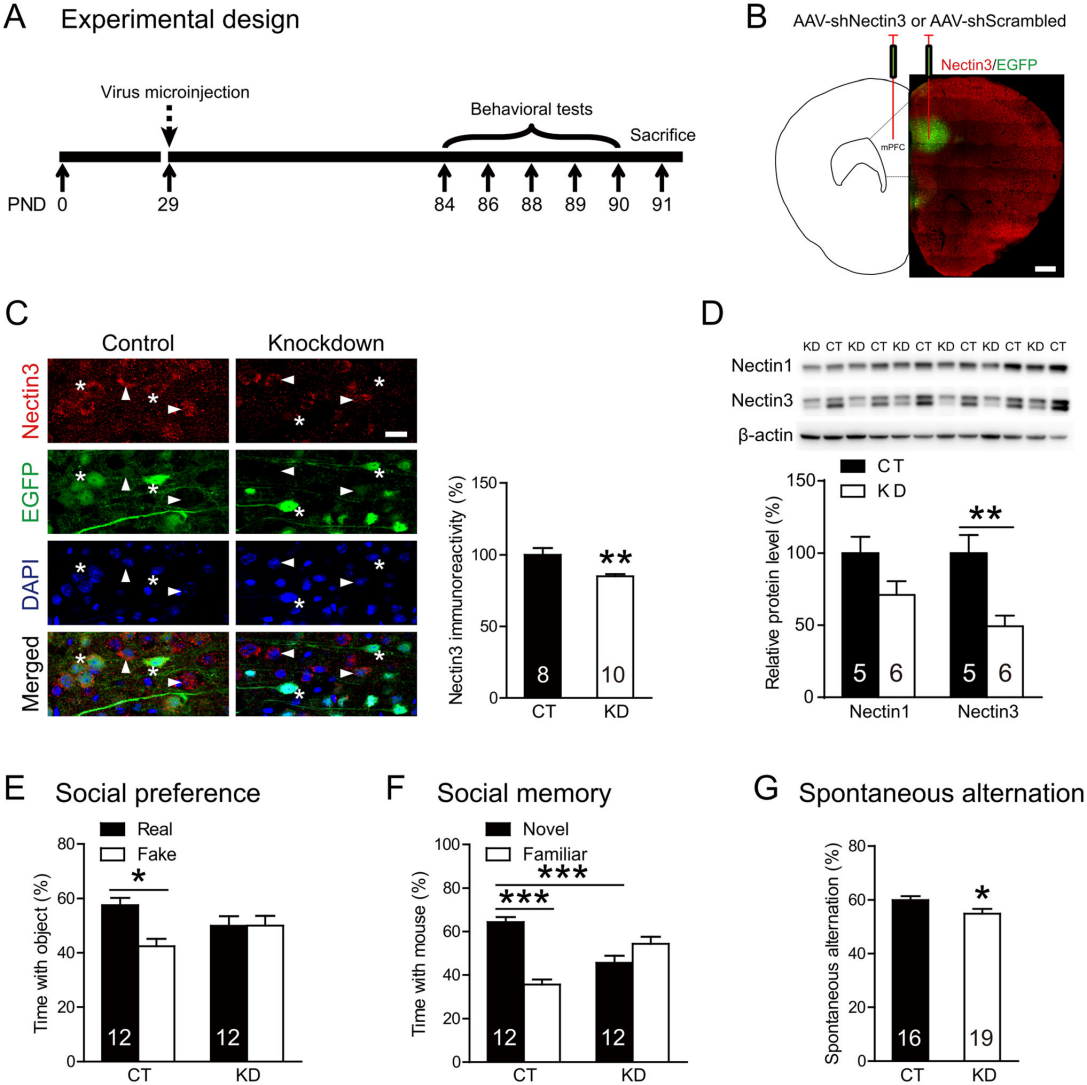
Behavioral effects of adolescent nectin3 knockdown in the medial prefrontal cortex (mPFC) of adult mice. **A** The experimental timeline of the behavioral procedure and brain tissue acquisition after virus injection. **B** Left panel, schematic of AAV microinjection into the mPFC; right panel, an image showing region-specific expression of EGFP in the mPFC (scale bar, 500 μm). **C** Representative images showing the expression of nectin3, EGFP, and DAPI in the mPFC of control and nectin3-KD mice (scale bar, 20 μm). **D** Western blot analyses confirm the knockdown-induced reduction of nectin3, but not nectin1, expression in the mPFC. **E** In the social preference test, unlike control mice that spend significantly more time with the real than the fake mice, nectin3-KD mice spend similar time interacting with fake and real mice. **F** In the social memory test, compared with control mice that successfully distinguish the novel from the familiar mouse, nectin3-KD mice show impaired social memory. **G** In the Y-maze spontaneous alternation task, nectin3-KD mice show impaired spatial working memory. CT, control; KD, knockdown. Numbers in each bar indicate the number of animals in each group. Data represent the mean ± SEM. *P < 0.05; **P < 0.01; ***P < 0.001.

Considering that stress-induced dendritic changes were evident throughout the mPFC, we injected AAV-shNectin3 into the mPFC (infection rate: 62.14%) to knock down nectin3 levels in adolescent mice (29 days old) without focusing on specific subregions and examined its impact at the same time point as in the stress experiment (day 84, Figs. 5A, B, and S9A). Compared with the controls, nectin3-KD mice showed significantly lower nectin3 immunoreactivity in the mPFC (*t*_(16)_ = 3.341, *P* = 0.004, Fig. 5C) and relatively unaltered nectin1 immunoreactivity levels (Fig. S9B). Western blot analyses further confirmed the reduction of nectin3, and not nectin1, expression levels (nectin3: *t*_(9)_ = 3.338, *P* = 0.008; nectin1: *t*_(9)_ = 1.874, *P* = 0.090; Fig. 5D).

The nectin3-KD and control mice exhibited comparable behaviors in the open field and social approach tests (all *P* > 0.299, Fig. S9C–D). Nevertheless, the nectin3-KD mice spent similar times interacting with the fake and real mice, which indicates impaired social preference (KD, *t*_(11)_ = 0.014, *P* = 0.989; CT, *t*_(11)_ = 2.806, *P* = 0.017, Figs. 5E and S9E). Nectin3-KD mice also failed to discriminate the novel from the familiar mouse in the social memory test (*t*_(16)_ = 1.332, *P* = 0.210, Fig. 5F), and their discrimination indices were significantly lower than those of controls (*t*_(22)_ = 4.645, *P* < 0.001), even though they showed a longer exploration time during the acquisition phase (Fig. S9F). In the Y-maze task, nectin3-KD mice had a significantly lower spontaneous alternation ratio than controls (*t*_(33)_ = 2.145, *P* = 0.039, Fig. 5G), despite similar numbers of entries in the two groups (Fig. S9G), suggesting an impairment in spatial working memory.

**Fig. 6:**
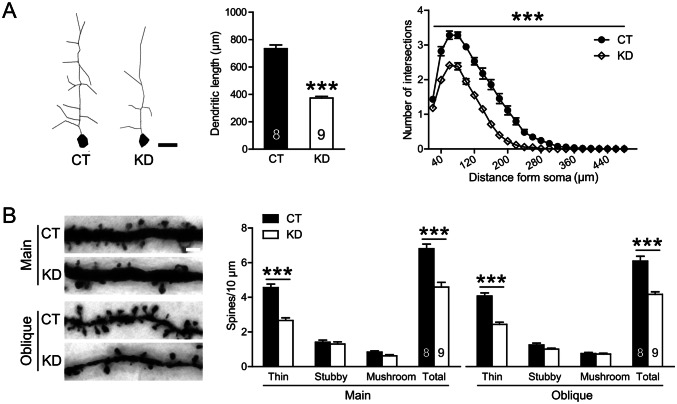
Morphological effects of adolescent nectin3 knockdown in the medial prefrontal cortex (mPFC) of adult mice. **A** Adolescent nectin3 knockdown reduces the length and complexity of the apical dendrites of pyramidal neurons (scale bar, 50 μm). **B** In nectin3-KD mice, the spine density on apical main and oblique dendrites is significantly reduced (scale bar, 20 μm). Six to eight neurons or dendritic segments were analyzed in the deep and superficial layers of three mPFC subregions. Morphological measures were averaged across layers and subregions for individual mice and then compared between groups. CT, control; KD, knockdown. Numbers in each bar indicate the number of animals in each group. Data represent the mean ± SEM. ***P < 0.001.

The morphological effects of prefrontal nectin3 knockdown during adolescence were largely consistent with the stress effects. Nectin3 knockdown decreased the length (*t*_(15)_ = 12.885, *P* < 0.001, Fig. 6A, middle panel) and complexity (*F*_(1, 15)_ = 116.200, *P* < 0.001, Fig. 6A, right panel) of the apical dendrites of mPFC pyramidal neurons, irrespective of the subregion (all *P* < 0.001, Fig. S10A–C) and layer (all *P* < 0.001, Fig. S10D–E). Nectin3 knockdown during adolescence also led to spine loss on the apical main and oblique dendrites in the mPFC (main: *t*_(15)_ = 5.798, *P* < 0.001; oblique: *t*_(15)_ = 6.333, *P* < 0.001; Fig. 6B). Detailed analyses showed that nectin3 knockdown specifically downregulated the number of thin spines (main: *t*_(15)_ = 7.610, *P* < 0.001; oblique: *t*_(15)_ = 7.568, *P* < 0.001), leaving stubby and mushroom spines relatively unaffected (all *t*_(15)_ < 2.107, all *P* > 0.052).

## Discussion

In the present study, we investigated the involvement of CAMs in adolescent stress-induced alterations in mPFC-dependent behaviors and structural plasticity. We found that adolescent chronic social instability stress impaired social recognition and spatial working memory, and induced dendritic atrophy and spine loss in pyramidal neurons of the mPFC. Importantly, among the several CAMs tested, nectin3 may mediate these stress effects, as adolescent stress specifically downregulated nectin3 expression in the mPFC, which correlated with behavioral deficits. Furthermore, suppression of mPFC nectin3 levels during adolescence successfully reproduced the effects of stress. These results suggest that nectin3 plays an important role in adolescent chronic stress effects in the mouse PFC.

Our finding that adolescent social instability stress impaired social memory is consistent with previous work [28, 35]. This impairment cannot be explained by reduced sensitivity to social cues, as the stressed mice demonstrated clear social preference and social interactions similar to control mice. We also demonstrated for the first time that chronic social instability stress in adolescent mice impaired spatial working memory as measured by spontaneous alternation in the Y maze. Note that spontaneous alternation measures an animal’s natural tendency to alternate choices on successive trials, which depends on several factors such as the anxiety level and spatial memory capacity and is mediated by many brain areas including the PFC [36]. Future studies are needed to validate the deleterious effects of adolescent chronic stress on mPFC-mediated cognitive functions with other behavioral tasks such as temporal order memory and reversal learning tests. The behavioral deficits we found were significantly correlated with the deterioration in fur state resulting from stress exposure [27], which confirms the link between stressful experiences and behavioral deficits. We also found that social memory and spatial working memory were impaired following nectin3 knockdown in the adolescent mPFC. Together with social and attentional deficits following other types of adolescent chronic stressors [10], these results confirm that disrupting the adolescent brain has deleterious effects on the complex social and cognitive functions mediated by the mPFC.

Neuronal structural abnormalities may underlie synaptic plasticity changes and behavioral dysfunction [1, 37–39]. Previous studies have shown that the effects of adolescent chronic stress on prefrontal dendritic architecture vary with stressor type and other factors (for a review, see [8]). For instance, social isolation or chronic restraint stress leads to reductions in dendritic arborization [40], spine density [41], and apical dendritic length [42], whereas peer play deprivation by housing with adult females leads to increased dendritic length and complexity in the PFC [43, 44]. Here we showed that adolescent chronic instability stress simplifies the dendritic structure and reduces spine density in mouse mPFC. Given that peer play deprivation is often achieved by housing adolescent rodents with an adult female that is not socially threatening [43, 44], the results from studies adopting negative physical or social stressors (e.g., social instability in our study) are consistent in demonstrating that stressful life experiences during adolescence lead to a simplified dendritic structure of mPFC pyramidal neurons.

While the CAMs we examined have been found to be altered by postnatal or adult stress, only one study has focused on adolescent stress and the mPFC; it showed that a reduction in prefrontal neuroligin2 may mediate the attentional deficits induced by chronic exposure to fear-inducing stressors on postnatal days 28–42 [10]. Our findings point to the involvement of nectin3 in adolescent stress-induced alterations of social and spatial working memory and prefrontal dendritic architecture. Both studies highlight synaptic CAMs as potential targets of adolescent chronic stress in the rodent mPFC, but we did not find significant changes in neuroligin2 following long-term social instability stress. Given that several aspects of the stress paradigms differ between our study and the previous report (e.g., stress duration, stressor type, animal species), future studies are required to explicitly test whether and how the PFC responds to different stressors in different ways.

Nectin3 is a Ca^2+^-independent immunoglobulin-like transmembrane protein that primarily anchors to the postsynaptic membrane at puncta adherentia junctions (PAJs)—the mechanical adhesion sites for neurotransmission [45]. Nectin3 forms heterophilic adhesions with presynaptic nectin1 and connects to the actin cytoskeleton *via* afadin, an actin filament-binding protein [46]. Nectin3 knockout has been shown to reduce the number of PAJs at mossy fiber–CA3 synapses and to change mossy fiber trajectories [47]. Our previous studies have linked hippocampal nectin3 to early-life stress-induced spine loss and cognitive deficits [16, 48]. As the first study to examine nectin3 in the PFC, we first confirmed that nectin3 is expressed in both excitatory and inhibitory neurons in mouse mPFC, similar to our findings in the hippocampus [30]. How nectin3 contributes to the stress effects on the neurodevelopment of the adolescent PFC is unknown. One possibility is that when nectin3 is downregulated by stress, the hetero-trans-dimers that it forms with nectin1 may be replaced by homo-trans-dimers, which have a lower affinity [49] and may result in weaker forms of PAJs and lead to the dying-back of dendrites due to the loss of a greater number of spines. Nectin3 may interact with other systems in the stress-induced effects on the structural development of the mPFC, such as the corticotropin-releasing hormone (CRH)-CRH receptor 1 signaling system [16, 50, 51], or other CAMs such as N-cadherins [52].

We also found some differences between adolescent stress and nectin3 knockdown. For example, nectin3 knockdown did not increase the anxiety-like behaviors as adolescent stress did. One explanation for these differences may be that the regions affected in nectin3 knockdown and adolescent stress were different: while nectin3 knockdown was confined to the PFC, adolescent stress experiences may affect multiple stress-related regions such as the hippocampus [27], which may underlie the increased anxiety-like behaviors induced by chronic stress. As our study primarily focused on the mPFC, future studies are warranted to investigate the effects of adolescent chronic stress on other stress-related regions and mPFC-related circuits [27, 53]. Structural plasticity was also altered differently by nectin3 knockdown and adolescent stress: while nectin3 knockdown caused a specific loss of thin spines, adolescent stress affected multiple spine types. The selective effects of nectin3 knockdown on thin spines have also been reported in the dentate gyrus [48], which may be related to spine-specific structural or functional properties (e.g., thin spines have smaller postsynaptic densities [54]).

In summary, our results support the hypothesis that nectin3 is a potential modulator of adolescent stress effects on the dendritic plasticity of mPFC pyramidal neurons and mPFC-dependent social recognition and spatial working memory. A better understanding of these pathophysiological mechanisms may promote the development of therapeutic targets for stress-related mental disorders.

## Electronic supplementary material

Below is the link to the electronic supplementary material.

Supplementary material 1 (PDF 978 kb)
