# Supplementary material for: Prefrontal Nectin3 Reduction Mediates Adolescent Stress-Induced Deficits of Social Memory, Spatial Working Memory, and Dendritic Structure in Mice
Source: Neurosci Bull. 2020 May 8;36(8):860–74. doi: 10.1007/s12264-020-00499-2 (PMC7410914; doi:10.1007/s12264-020-00499-2)
Supplement: Supplementary file 1 — Supplementary material 1 (PDF 978 kb) [file 12264_2020_499_MOESM1_ESM.pdf]

Supplementary Information

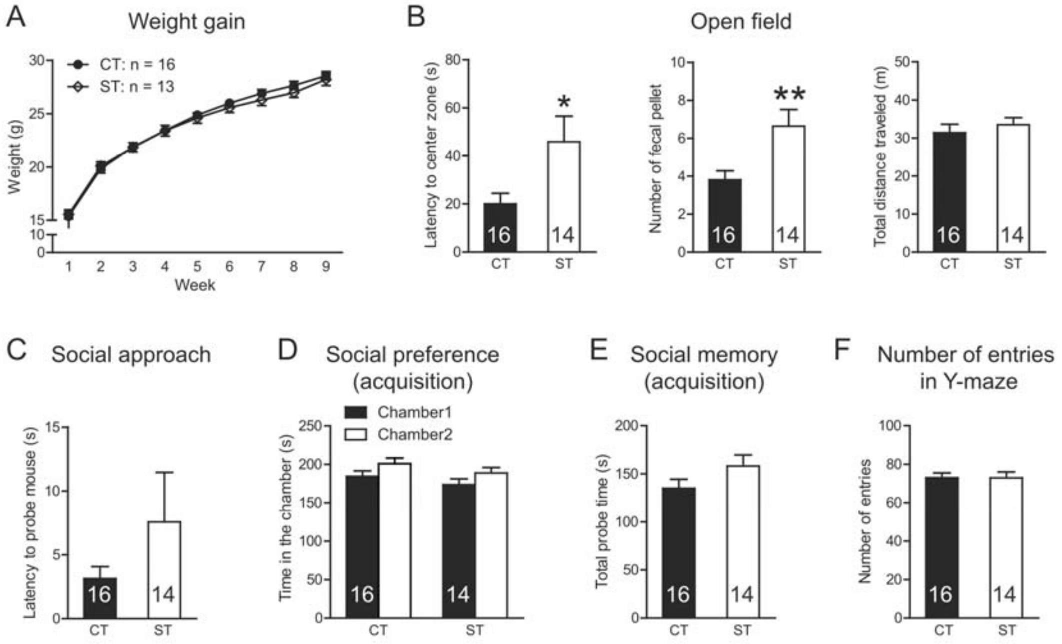

Fig. S1. Additional behavioral effects of adolescent chronic social instability stress. (A) The control and stressed mice showed comparable weight gain during and after stress treatment. ANOVA of body weight revealed a significant main effect of day ($F_{(8,\ 216)}$ = 963.983, P < 0.001), but not treatment ($F_{(1,\ 27)}$ = 0.177, P = 0.678) or treatment × day interaction ($F_{(8,\ 216)}$ = 1.569, P = 0.135). (B) In the open field test, adolescent stress significantly increased the latency to the center zone (($t_{(28)}$ = 2.244, P = 0.038) and the number of fecal pellet ($t_{(28)}$ = 2.941, P = 0.006), without altering the total distance traveled ($t_{(28)}$ = 0.712, P = 0.482). (C) In the social approach test, adolescent stress did not alter the latency to probe the target mouse ($t_{(28)}$ = 1.182, P = 0.247). (D) During the acclimation phase of the social preference test, control and stressed mice showed comparable time in the two chambers (CT: $t_{(15)}$ = 1.383, P = 0.187; ST: $t_{(13)}$ = 1.331, P = 0.206). (E) During the acquisition phase of the social memory test, control and stressed mice spent similar time interacting with the target mice ($t_{(28)}$ = 1.580, P = 0.125). (F) In the Y-maze test, stress did not affect the number of entries to three arms ($t_{(28)}$ = 0.002, P = 0.998). CT, control; ST, stress. Numbers in each bar indicate the number of animals in each group. Data represent mean ± SEM. * P < 0.05; ** P < 0.01.

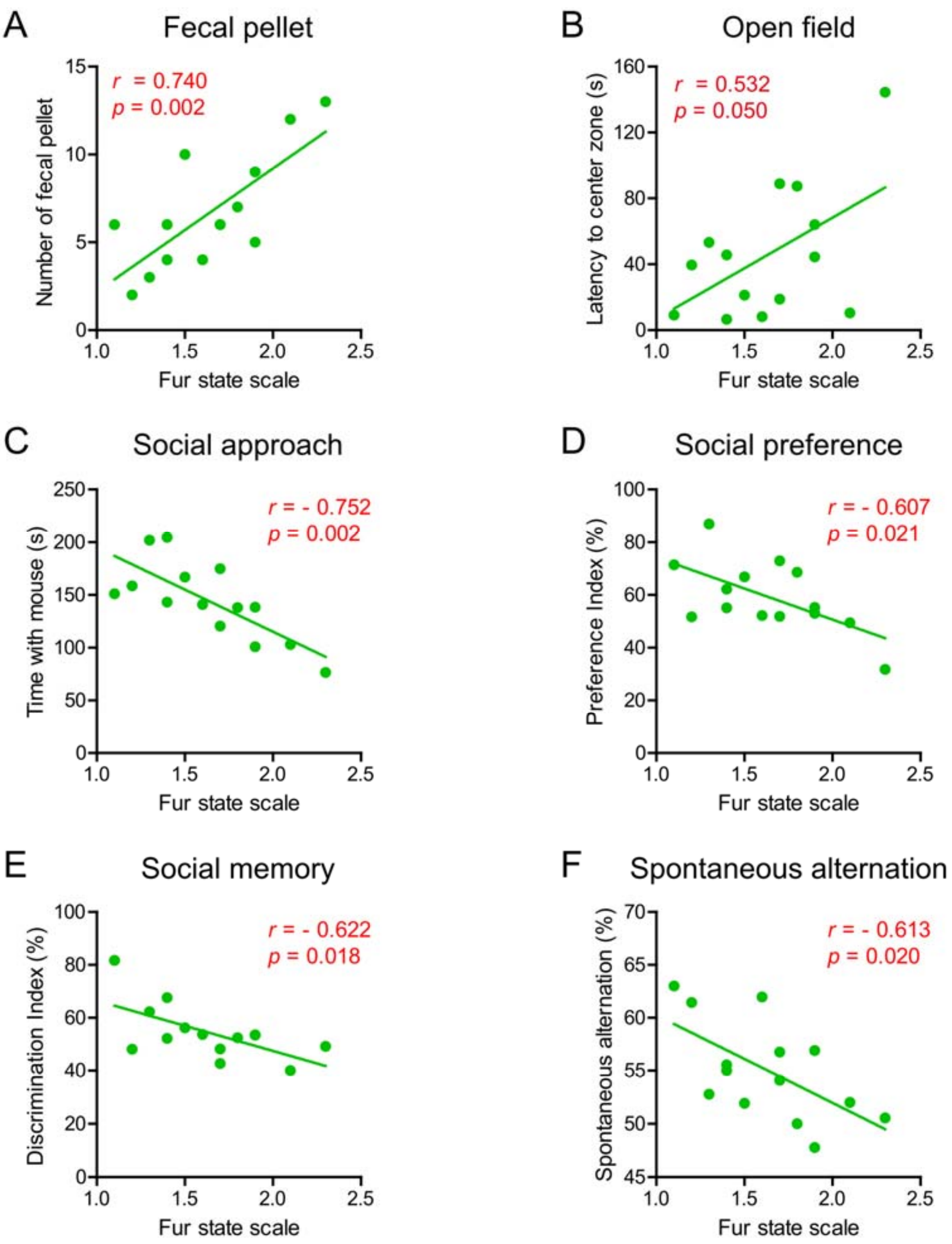

Fig. S2. Correlations between fur state and behavioral performances in stressed mice. (A) The number of fecal pellet and (B) latency to the center zone in the open field test; (C) Exploration time with mouse in the social approach test; (D) Social preference index; (E) Discrimination index in the social memory test; (F) Spontaneous alternation ratio in the Y maze.

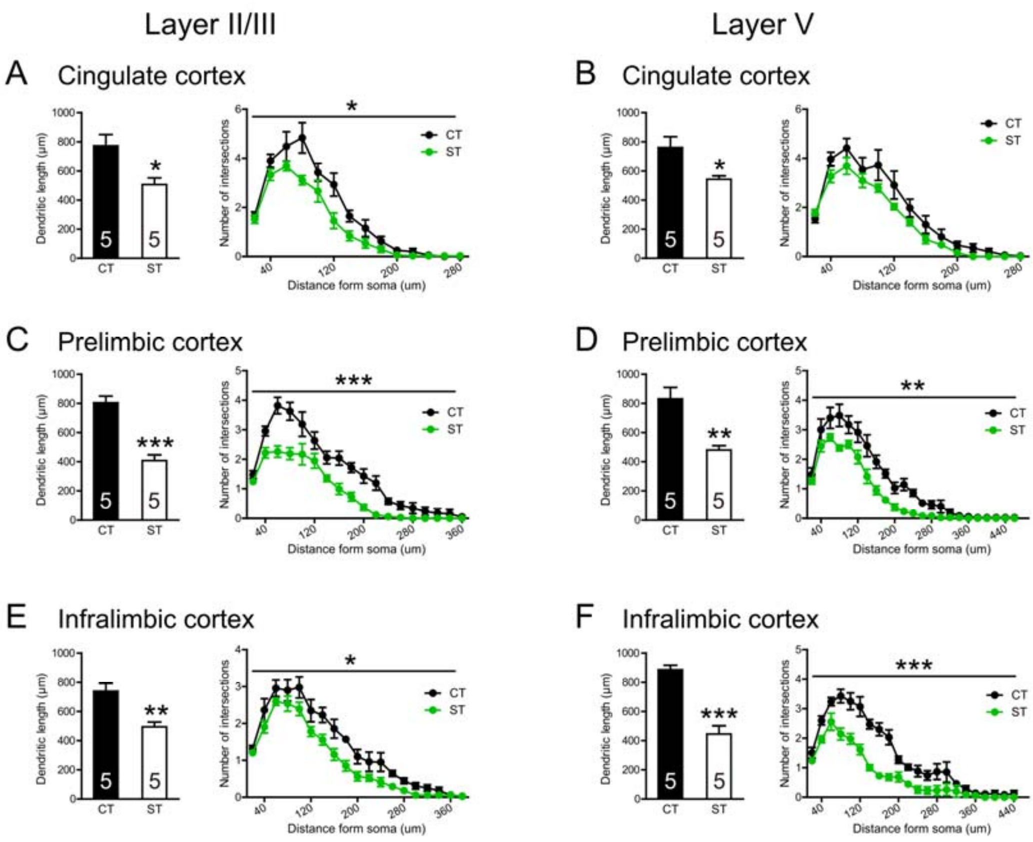

Fig. S3. Effects of adolescent chronic social instability stress on dendritic architecture in superficial and deep layers of medial prefrontal cortex (mPFC) subregions. Compared with control mice, except for the number of intersections in the layer V of cingulate cortex (B), adolescent stress reduced the length and complexity of apical dendrites of pyramidal neurons in layers II/III and V of cingulate cortex (A-B), prelimbic cortex (C-D), and infralimbic cortex (E-F). CT, control; ST, stress. Numbers in each bar indicate the number of animals in each group. Data represent mean ± SEM. * $P < 0.05$; ** $P < 0.01$; *** $P < 0.001$.

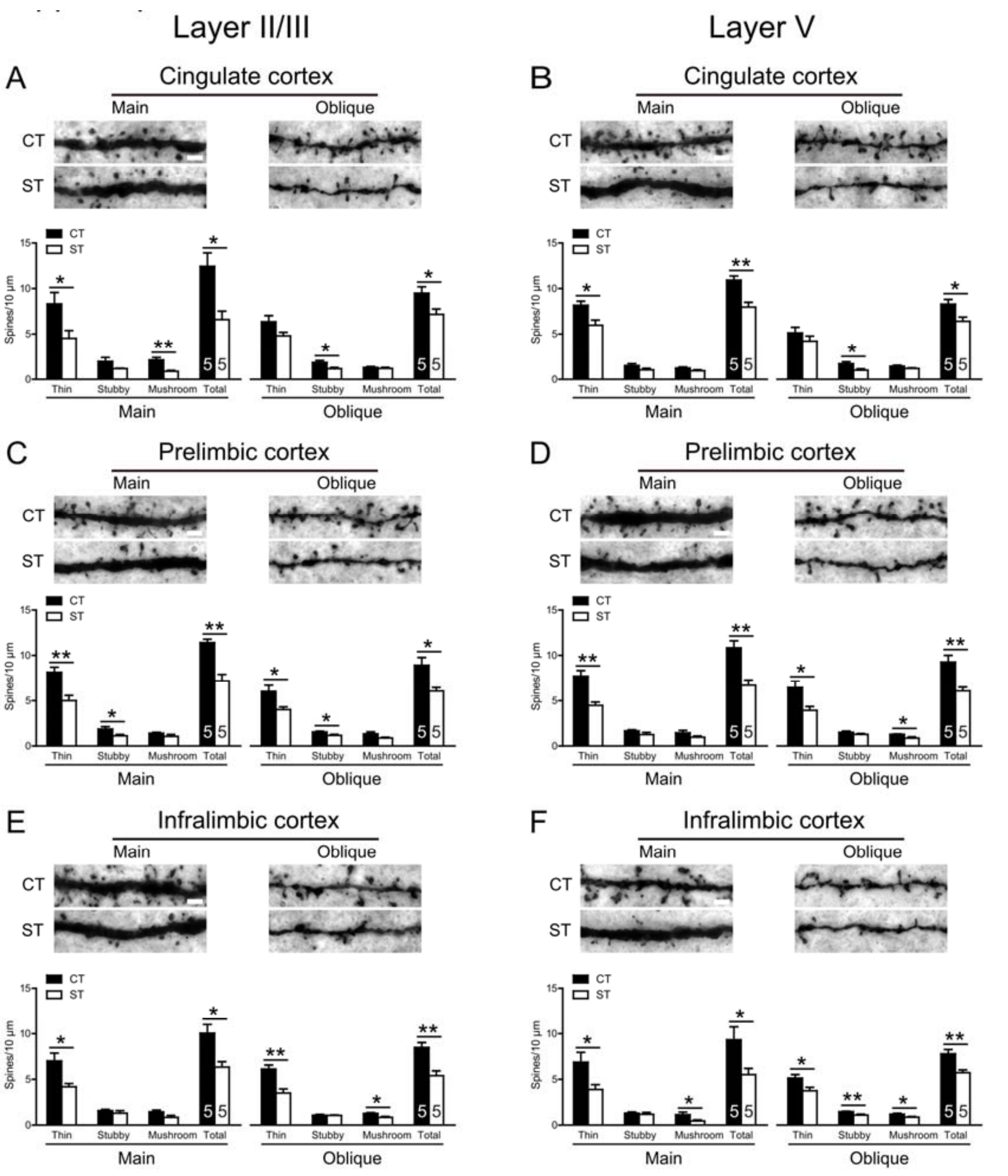

Fig. S4. Effects of adolescent chronic social instability stress on spine density in superficial and deep layers of medial prefrontal cortex (mPFC) subregions. Compared with control mice, adolescent stress reduced the number of spines in apical main and oblique dendrites in layers II/III and V of cingulate cortex (A-B), prelimbic cortex (C-D), and infralimbic cortex (E-F). CT, control; ST, stress. Numbers in each bar indicate the number of animals in each group. Data represent mean ± SEM. * $P < 0.05$; ** $P < 0.01$.

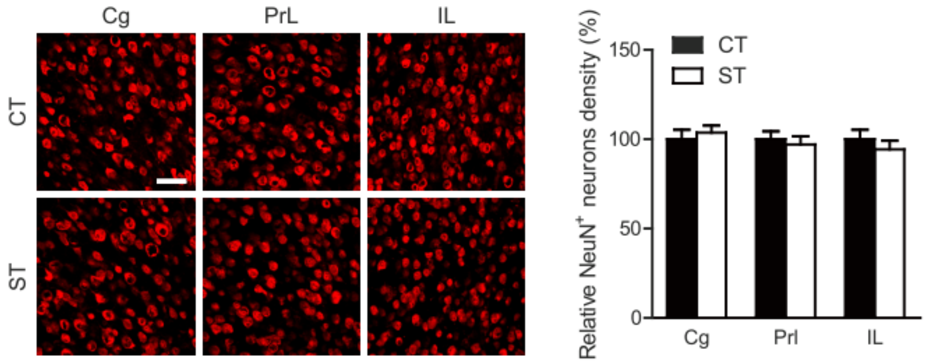

Fig. S5. Effects of adolescent chronic social instability stress on the neuronal number in mouse medial prefrontal cortex (mPFC). Control and stressed mice had comparable density of neuronal nuclei antigen (NeuN)-positive neurons in three subregions of mPFC. Scale bar = 50 μm. Cg, cingulate cortex; CT, control; IL, infralimbic cortex; PrL, prelimbic cortex; ST, stress. Data represent mean ± SEM.

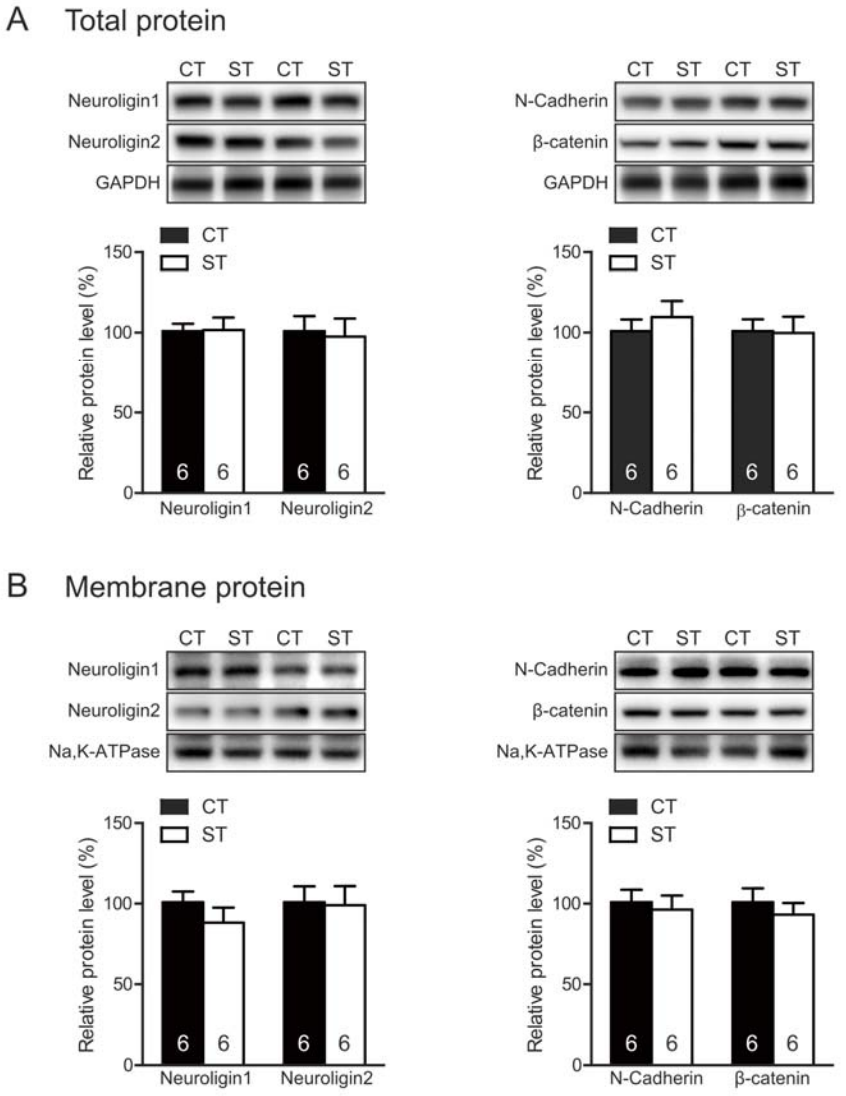

Fig. S**6.** Additional effects of adolescent chronic social instability stress on neural cell adhesion molecules in mouse medial prefrontal cortex (mPFC). Adolescent stress did not affect the total (A, all $t_{(10)} < 0.719$, all $P > 0.489$) or membrane (B, all $t_{(10)} < 1.103$, all $P > 0.296$) protein levels of neuroligin1, neuroligin2, N-Cadherin, or β-catenin in mouse mPFC. CT, control; ST, stress. Numbers in each bar indicate the number of animals in each group. Data represent mean ± SEM.

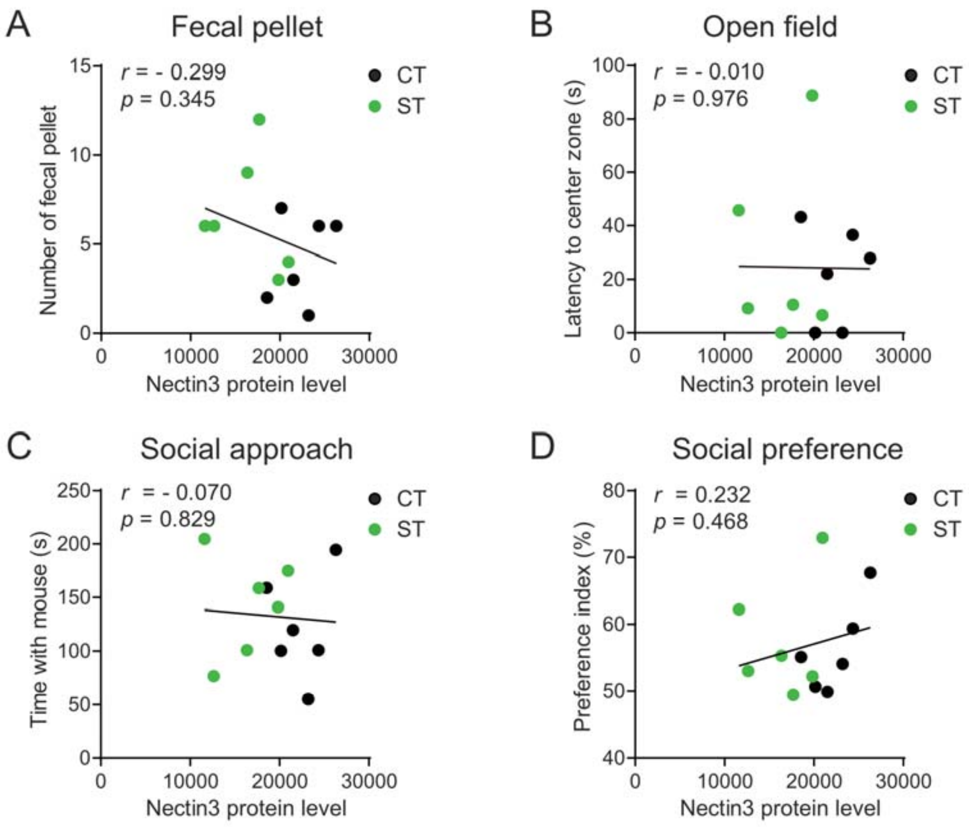

Fig. S7**.** The correlations between nectin3 membrane expression in medial prefrontal cortex and behavioral performances. (A) The number of fecal pellet and (B) latency to center zone in the open field; (C) Exploration time with mouse in the social approach test; (D) Social preference index. CT, control; ST, stress.

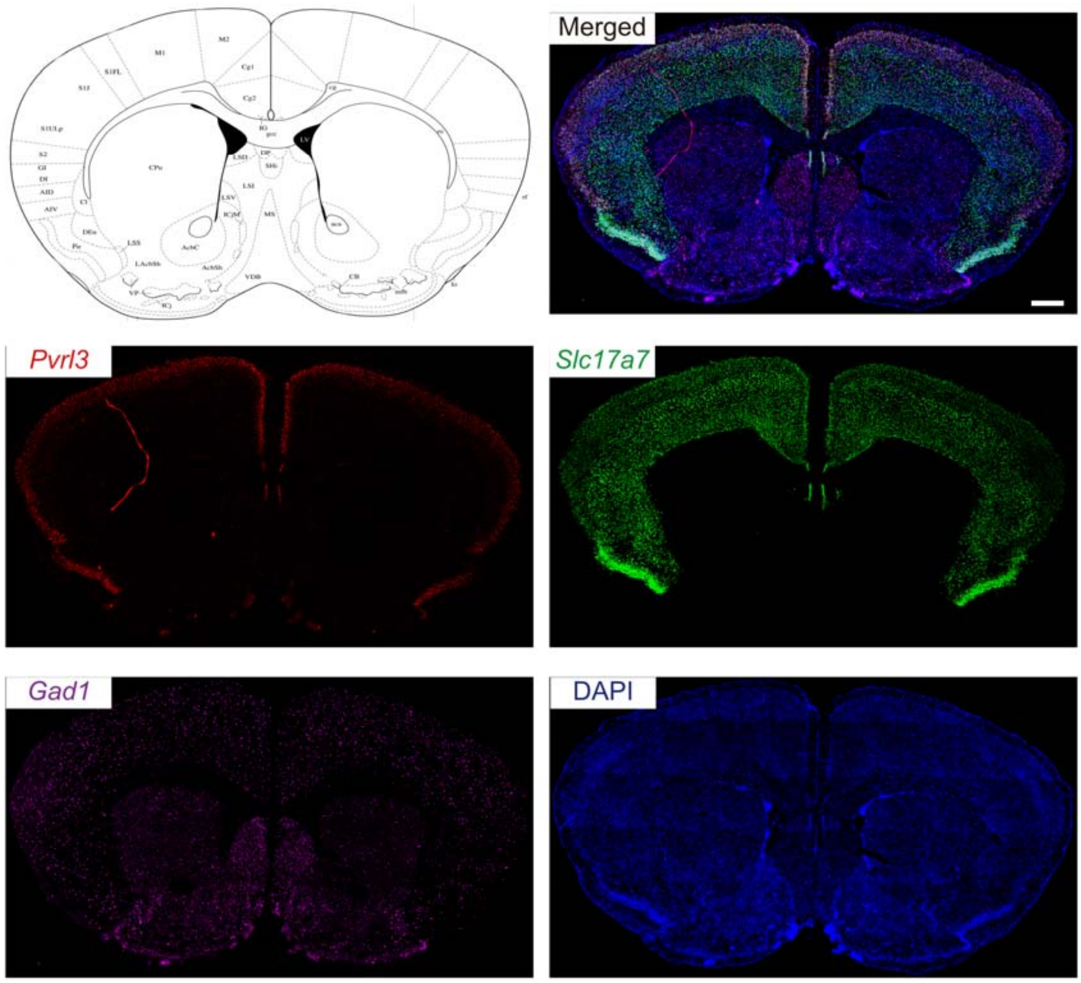

Fig. S8. Representative images showing the regional specificity of mRNA expression of *Pvrl3*, *Slc17a7,* and *Gad1* in the mouse brain (scale bar = 500 µm). Specifically, the VGluT1 mRNA, *Slc17a7,* was expressed in cortical, not subcortical, areas, whereas the mRNA of nectin3 (*Pvrl3*) and GAD67 (*Gad1*) were expressed in both cortical and subcortical areas, with nectin3 mRNA highly expressed in the superficial (II/III) layers.

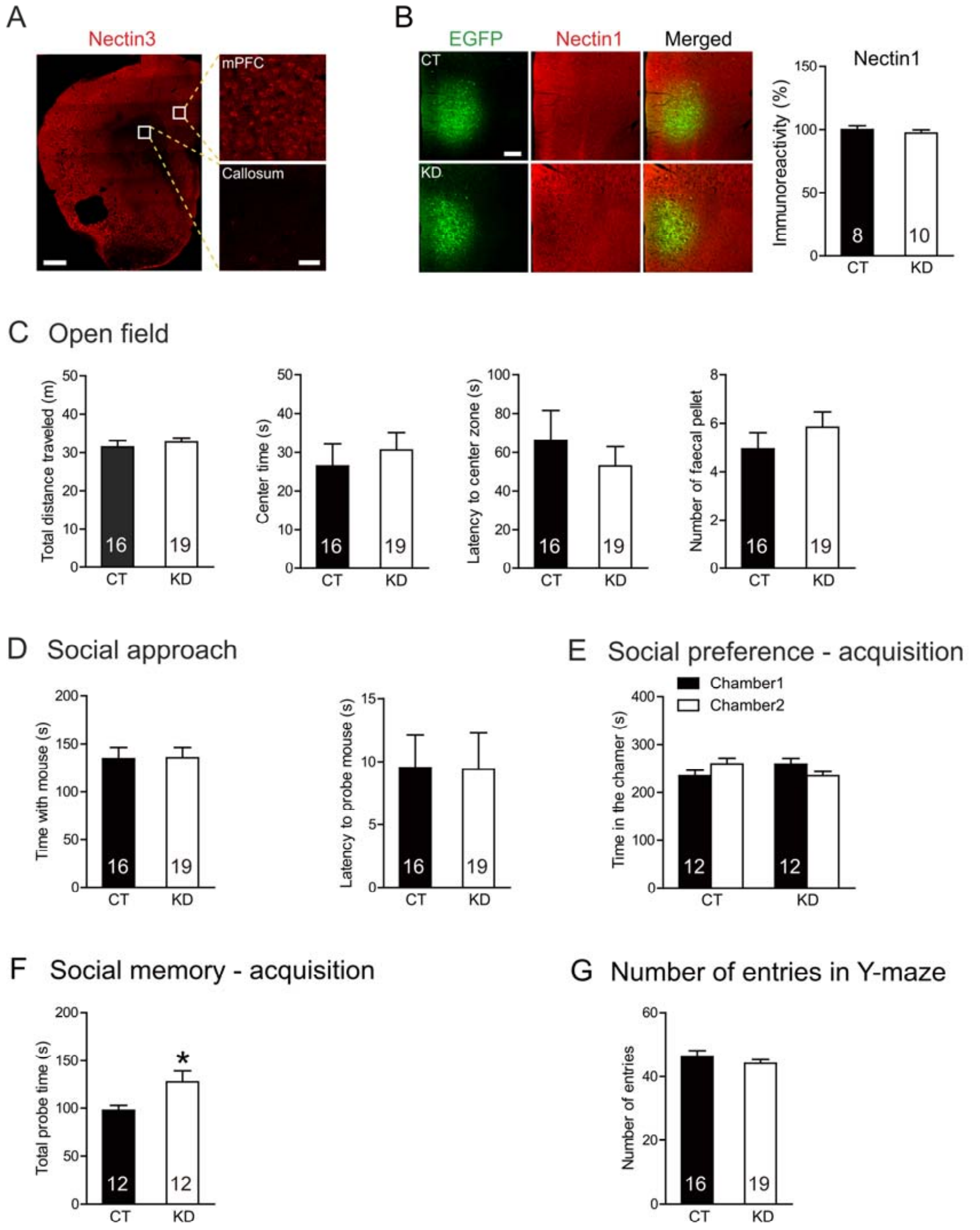

Fig. S**9.** Effects of adolescent nectin3 knockdown in medial prefrontal cortex (mPFC) on nectin1 expression and behaviors. (A) Representative images showing nectin3 expression in mouse brain (left: scale bar = 500 μm; right: scale bar = 50 μm). (B) Protein levels of nectin1 in mPFC remained unchanged in *Nectin3_KD* mice (scale bar = 200 μm). (C) In the open field test, *Nectin3_KD* mice showed similar anxiety levels with control mice (all $t_{(33)} < 0.738$, all $P > 0.466$). (D) Nectin3 knockdown did not alter the time spent with mouse ($t_{(33)} = 0.065$, $P = 0.948$) or the latency ($t_{(33)} = 0.028$, $P = 0.978$) to probe mouse in the social approach test. (E) *Nectin3_KD* and

control mice showed comparable exploration time in the acquisition session of the social preference test. (F) *Nectin3_KD* mice spent significantly more time interacting with stimulus mice in the acquisition session of the social memory test ($t_{(33)} = 2.445$, $P = 0.027$); (G) *Nectin3_KD* and control mice showed comparable number of entries in the Y-maze; CT, control; KD, knockdown. Numbers in each bar indicate the number of animals in each group. Data represent mean ± SEM. * $P < 0.05$.

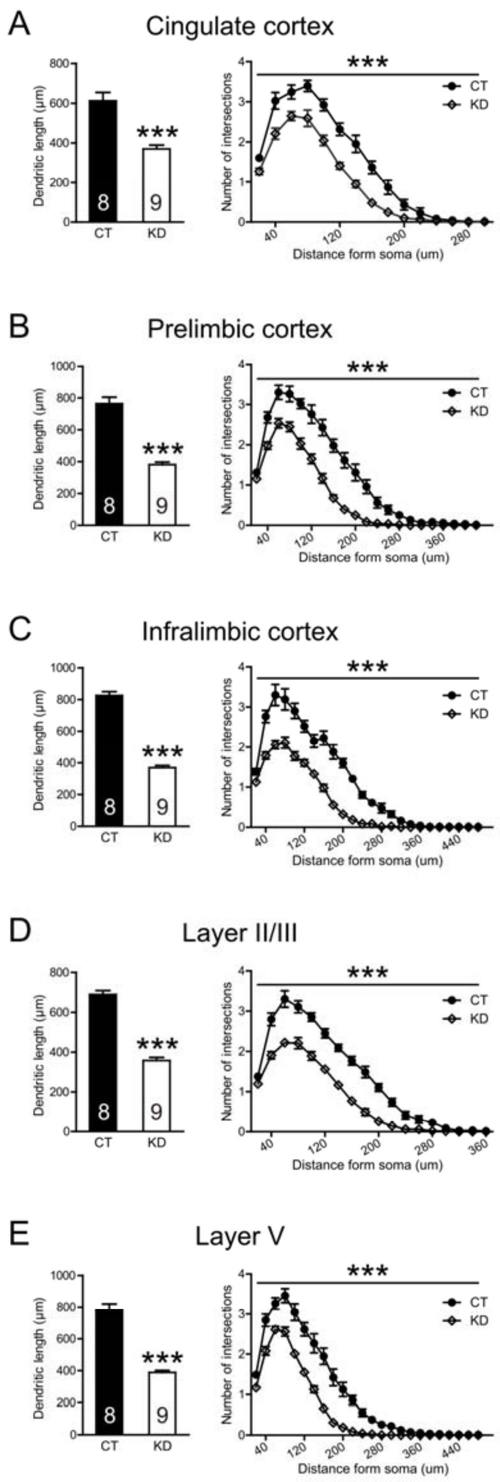

Fig. S10. Effects of adolescent nectin3 knockdown on dendritic architecture in mPFC subregions and layers. Nectin3 knockdown in mPFC reduced the length and complexity of apical dendrites in cingulate (A), prelimbic (B), and infralimbic (C) regions, and in layers II/III (D) and layer V (E). CT, control; KD, knockdown. Numbers in each bar indicate the number of animals in each group. Data represent mean ± SEM. *** $P < 0.001$.
